# Mapping the dengue scientific landscape worldwide: a bibliometric and network analysis

**DOI:** 10.1590/0074-02760160423

**Published:** 2017-03-27

**Authors:** Fabio Batista Mota, Bruna de Paula Fonseca e Fonseca, Andréia Cristina Galina, Roseli Monteiro da Silva

**Affiliations:** 1 Fundação Oswaldo Cruz-FiocruzRio de JaneiroRJBrasilFundação Oswaldo Cruz-Fiocruz, Vice-Presidência de Pesquisa e Laboratórios de Referência, Rio de Janeiro, RJ, Brasil; 2 Fundação Oswaldo Cruz-FiocruzCentro de Desenvolvimento Tecnológico em SaúdeRio de JaneiroRJBrasilFundação Oswaldo Cruz-Fiocruz, Centro de Desenvolvimento Tecnológico em Saúde, Rio de Janeiro, RJ, Brasil

**Keywords:** dengue, scientific publications, scientific landscape, bibliometrics, social network analysis

## Abstract

**BACKGROUND:**

Despite the current global trend of reduction in the morbidity and mortality of neglected diseases, dengue’s incidence has increased and occurrence areas have expanded. Dengue also persists as a scientific and technological challenge since there is no effective treatment, vaccine, vector control or public health intervention. Combining bibliometrics and social network analysis methods can support the mapping of dengue research and development (R&D) activities worldwide.

**OBJECTIVES:**

The aim of this paper is to map the scientific scenario related to dengue research worldwide.

**METHODS:**

We use scientific publication data from Web of Science Core Collection - articles indexed in Science Citation Index Expanded (SCI-EXPANDED) - and combine bibliometrics and social network analysis techniques to identify the most relevant journals, scientific references, research areas, countries and research organisations in the dengue scientific landscape.

**FINDINGS:**

Our results show a significant increase of dengue publications over time; tropical medicine and virology as the most frequent research areas and biochemistry and molecular biology as the most central area in the network; USA and Brazil as the most productive countries; and Mahidol University and Fundação Oswaldo Cruz as the main research organisations and the Centres for Disease Control and Prevention as the most central organisation in the collaboration network.

**MAIN CONCLUSIONS:**

Our findings can be used to strengthen a global knowledge platform guiding policy, planning and funding decisions as well as to providing directions to researchers and institutions. So that, by offering to the scientific community, policy makers and public health practitioners a mapping of the dengue scientific landscape, this paper has aimed to contribute to upcoming debates, decision-making and planning on dengue R&D and public health strategies worldwide.

Dengue is an acute febrile disease transmitted by the mosquito-borne dengue virus. It is a self-limiting illness, characterised by fever, myalgia, headache and rash, and its severe forms (hemorrhagic fever and shock syndrome) may lead to multisystem involvement and death (National Centre for Biotechnology Information: ncbi.nlm.nih.gov/mesh/68003715). Dengue’s incidence and mortality have been increasing in recent years and areas of occurrence have expanded worldwide - currently more than 125 countries are considered dengue endemic (WHO 2009, Bhatt et al. 2013, Murray et al. 2013). Annual estimates of dengue infections range from 50 to 200 million cases and vector control remains the most important way to prevent and control the disease (Murray et al. 2013). Although its global impact is difficult to estimate because of inadequate disease surveillance, misdiagnosis and low levels of reporting, it has great potential impact on public health worldwide due to rapid epidemic spread beyond national borders (WHO 2009).

Dengue is also a scientific and technological challenge since there is no specific treatment or effective vaccine, vector control or public health intervention. In this context, bibliometric analysis can support the mapping of research and development (R&D) activities, characterising and quantifying the scientific output of dengue research. Bibliometrics involves the application of mathematical and statistical methods to the analysis of scientific publications available in journal-indexing databases, such as the Web of Science (WoS). In recent years, bibliometric analysis has been widely conducted to evaluate scientific research activities in other arboviral diseases such as chikungunya (Vera-Polania et al. 2015), malaria (Muñoz-Urbano et al. 2014), yellow fever disease (Bundschuh et al. 2013) and Zika virus (Martinez-Pulgarin et al. 2016).

Although a few studies have addressed dengue research bibliometrics worldwide (Dutt et al. 2010, Bhardwaj 2014, Thirumagal & Nehru 2015), none of them take advantage of social network analysis (SNA) techniques to go beyond traditional indicators to evaluate research output. SNA and co-authorship networks are being increasingly used as powerful tools to assess collaboration trends and to identify leading scientists and organisations in health studies (Fonseca et al. 2016). The analysis reveals the social structure of the networks by identifying actors (researchers, organisations, countries etc.) and their connections and can be applied to strengthen the collaboration between network members or to assess network functioning (Kothari et al. 2014). Important public health issues can be addressed by SNA, including disease transmission, information diffusion (e.g., diffusion of innovations) and analyses related to the interorganisational structure of health systems (Luke & Harris 2007).

From this perspective, the aim of this paper is to map the scientific scenario related to dengue research worldwide, combining bibliometrics and SNA techniques. The paper joins two substantial sets of information. The first describes the comprehensive research status of the dengue field by analysing the quantity of publications, main journals, frequent research areas, most cited papers and most scientifically productive countries and organisations. The second explores the global cooperation network of these organisations, identifying most central players, and the association of different research topics, highlighting the one that is mostly associated with knowledge generation in the field. The information presented herein aims to generate evidence that could ultimately inform managers, researchers and policy makers, supporting decision making, R&D planning and financing strategies.

## MATERIALS AND METHODS

*Data retrieval* - Bibliometric and SNA techniques were combined to generate qualified information related to dengue from scientific publications indexed at Thomson Reuters WoS Core Collection. The search was carried out on October 2015, encompassing the years of 1945 to 2014. The search was carried out on advanced search mode using the Topic (ts) search field, which encompasses title, abstract and key words. In WoS, key words include authors’ key words and key words plus, which are attributed to the articles by WoS editors after reviewing the titles of the article’s references, thus broadening search results. The search strategy was restricted to retrieve only articles indexed in SCI-EXPANDED. The search query used was: [ts = (dengue*)] and Document Types: (Article), Indexes = SCI-EXPANDED, Timespan = 1945-2014.

The choice of analysing only articles was justified by the high standards required for publication on periodicals (double blind peer review, usually). Not only are they considered more complete, but also correspond to more advanced stages of research compared, for instance, to papers published on meetings proceedings (González-Albo & Bordons 2011).

*Standardisation and cleaning the data* - Retrieved data (n = 10,043) were imported from the WoS as raw data files in plain text format into the data/text mining software VantagePoint 9.0 (Search Technology Inc). The following procedures were adopted prior to analysis: (a) duplicates were removed using ISI Unique Article Identifier; (b) records directly related to dengue were retrieved by searching for the descriptor “dengue” in a new field of analysis that merged only the fields Title, Abstract and Author’s key words (n = 8,514); and (c) the fields “author affiliations (Organisation and City and Country)” and “country” were normalised using the general fuzzy logic from VantagePoint’s list cleanup tool as well as manual cleaning.

*Analysis, network assembly and visualisation* - Cleaned and processed data regarding rankings for countries, journals, cited references, and organisations were produced on VantagePoint and exported to Microsoft Excel for graphical representation. These data referred to the period 1990-2014, which corresponded to 7,790 records. The full period data collection had the specific aim of illustrating the evolution of the publications on dengue since the beginning of the SCI-EXPANDED index.

For network assembly and analysis, after standardisation and cleaning, co-occurrence matrices were produced to generate (a) organisational networks, based on the “author affiliations (Organisation and City and Country)” field, and (b) research area networks, based on the homonym field. The open source software Gephi 0.8.2 was used to visualise the network graphs and perform the statistical analysis. Networks were generated using the Force Atlas 2 algorithm.

The most central research areas and organisations in their respective networks were identified by calculating their degree centrality. Degree centrality is based on the number of a node’s (organisation or research area) direct connections to other nodes in the network, being a highly effective measure of prominence and influence (Freeman 1979). The organisations or research areas with high degree centrality have strategic significance in the network.

The timespan for network analysis covered the period 2000-2014, corresponding to 6,952 records. This 15-year cumulative approach represented a historical outlook of the collaboration for research in dengue and has been adopted in previous studies (Vasconcellos & Morel 2012). The cumulative networks are an indication of the ever-growing underlying social network that potentially functions as a network through which relevant innovation-related knowledge can persist (Breschi & Lissoni 2005).

*Limitations* - It is possible to have articles included in the dataset despite presenting an investigation focus that is not directly related to the theme of analysis. Nonetheless, such possibility is always present in bibliometric analyses. This holds true even when all retrieved documents undergo a manual selection (usually by reading the title and abstract) to exclude those not directly related to the purpose of the analysis, since the inclusion/exclusion decision is also subjective.

## RESULTS AND DISCUSSION

**Fig. 1:**
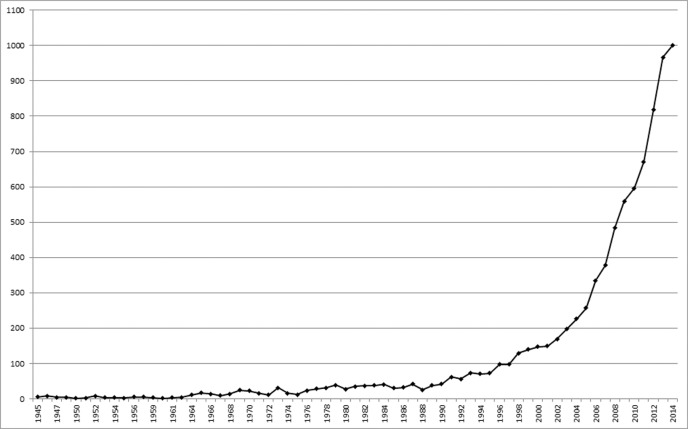
: evolution of world scientific publications on dengue (1945-2014).

Fig. 1 shows the evolution of scientific publications related to dengue worldwide. From 1990 to 2014 the scientific publications increased 2,280%, surpassing the symbolic barrier of 100 articles per year in 1998. Over the decade 2005-2014, dengue publications grew 289%, reaching a peak of 1,000 articles in the last year of the period. This increase in dengue research could have been fostered by the rapid geographical expansion of dengue and the subsequent increase in R&D funding (Bhatt et al. 2013, Horstick et al. 2015). Many dengue-endemic countries have experienced rapid economic growth and the imminent threat of dengue to noninfected areas, including Western countries, has risen interests of novel actors from both the academic and corporate world. Large-scale funding agencies have supported several projects for dengue R&D in the last ten years, and in 2012 dengue was the fourth highest funded neglected tropical disease globally following HIV/AIDS, malaria, and tuberculosis, with an investment of nearly a quarter billion dollars (Horstick et al. 2015).

The significant increase in the number of publications from 1990 onwards probably occurred due to the indexing of articles’ Abstracts in the WoS database, which started in this same year. For this reason, if a search for articles published between 1945 and 1989 was carried out in the WoS Title field, it would have returned virtually identical results to the same search in the Topic field, though the latter included title, abstract and key words. During this period, differences in the results obtained by searching the Topic or Title field were minimal. When they occurred, this was most likely due to occasional indexing revision of articles published before 1990, after the inclusion of their Abstract on the WoS database.

**Fig. 2:**
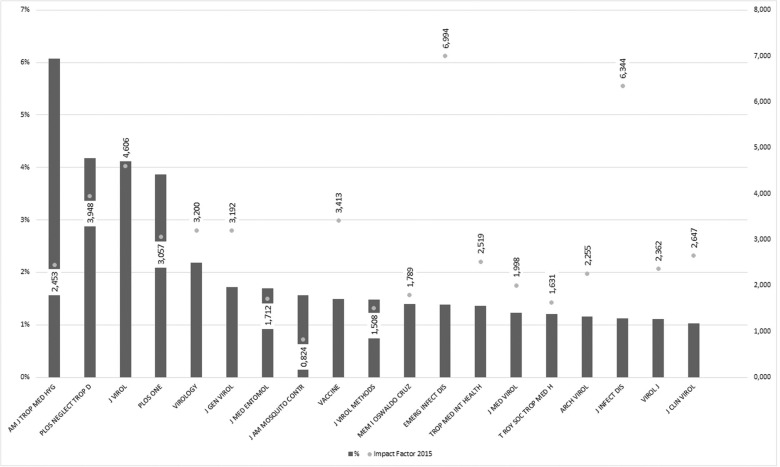
: most frequent journals in scientific publications on dengue (1990-2014). *American Journal of Tropical Medicine and Hygiene; PLoS Neglected Tropical Diseases; Journal of Virology; PLoS ONE; Virology; Journal of General Virology; Journal of Medical Entomology; Journal of the American Mosquito Control Association; Vaccine; Journal of Virological Methods; Memórias do Instituto Oswaldo Cruz; Emerging Infectious Diseases; Tropical Medicine & International Health; Journal of Medical Virology; Transactions of The Royal Society of Tropical Medicine and Hygiene; Archives of Virology; Journal of Infectious Diseases; Virology Journal; Journal of Clinical Virology.

Fig. 2 shows the main scientific journals publishing research on dengue and their impact factors. The journals were ranked according to the frequency of published articles, indicating those that were mainly chosen by the dengue scientific community to disseminate research. The three most frequent journals are the American Journal of Tropical Medicine and Hygiene (6.07%), PLoS Neglected Tropical Diseases (4.17%) and Journal of Virology (4.12%). Journals with a more general profile, such as PLoS ONE (3.86%) and Memórias do Instituto Oswaldo Cruz (1.40%) were also present. Memórias is a Brazilian journal owned by Fundação Oswaldo Cruz (Fiocruz), a public health institution linked to the Brazilian government. The presence of a Brazilian journal in this rank was probably due to the role of dengue as a critical public health problem in the country and to the weight of Fiocruz on dengue scientific landscape as we will see further. The presence of a vaccine specialised journal, accounting for 1.49% of the scientific publications, probably reflects the large volume of R&D investments worldwide with the aim of obtaining dengue-specific vaccines. The key word ‘vaccine’ was one of the most frequent on dengue publications, included in 3% of the publications between 1990 and 2014. In 2012, about 68% of dengue R&D funding came from the pharmaceutical industry - with most of this funding probably allocated for dengue vaccine development - whereas its share was 24% in 2007 (Horstick et al. 2015). A more general discussion on dengue vaccines can be found in the work of Thisyakorn and Thisyakorn (2013).

Table depicts the most frequently cited references in scientific publications on dengue (references with at least 240 citations). The percentage corresponds to the ratio between the number of citations and the total number of articles in the period 1990-2014 (7,790). The top three cited references were Gubler (1998), on the changing epidemiology of dengue and dengue hemorrhagic fever, Halstead (1988), on the structure of dengue genomes as a means to understand the pathogenetic mechanisms of the disease, and Lanciotti et al. (1992), on the development and application of a rapid assay for detecting and typing dengue viruses. A joint publication between the World Health Organization (WHO) and the Special Programme for Research and Training in Tropical Diseases (TDR) (WHO 2009) was the most cited reference under ten years of publication. This publication aimed to contribute to prevention and control of dengue morbidity and mortality and also to serve as a guide to health practitioners and researchers worldwide. In a certain extent, the publications showed in Table can be considered the most relevant studies on dengue. Thus, based on their percentage of citations, it is fair to say that these publications are the basis for knowledge building in this broad field of investigation as well as public health interventions.

**TABLE:** Most cited references in scientific publications on dengue (1990-2014)

Title	Author (1st)	Pub. year	Source (Abbrev)	%
Dengue and dengue hemorrhagic fever	Gubler DJ	1998	CLIN MICROBIOL REV	10,98%
Pathogenesis of dengue: challenges to molecular biology	Halstead SB	1988	SCIENCE	8,00%
Rapid detection and typing of dengue viruses from clinical samples by using reverse transcriptase-polymerase chain reaction	Lanciotti RS	1992	J CLIN MICROBIOL	7,91%
Dengue hemorrhagic fever Diagnosis, treatment, prevention and control	WHO	1997	WHO	6,03%
Dengue viremia titer, antibody response pattern, and virus serotype correlate with disease severity	Vaughn DW	2000	J INFECT DIS	5,92%
Epidemic dengue/dengue hemorrhagic fever as a public health, social and economic problem in the 21st century	Gubler DJ	2002	TRENDS MICROBIOL	5,60%
Dengue: guidelines for diagnosis, treatment, prevention and control	WHO	2009	WHO	5,58%
Dengue: an update	Guzman MG	2002	LANCET INFECT DIS	5,02%
Dengue	Halstead SB	2007	LANCET	5,01%
Dengue: the risk to developed and developing countries	Monath TP	1994	P NATL ACAD SCI	4,84%
Flavivirus genome organization, expression, and replication	Chambers TJ	1990	ANNU REV MICROBIOL	4,56%
A prospective study of dengue infections in Bangkok	Burke DS	1988	AM J TROP MED HYG	4,03%
An enzyme-linked immunosorbent assay to characterize dengue infections where dengue and Japanese encephalitis co-circulate	Innis BL	1989	AM J TROP MED HYG	4,03%
Techniques for hemagglutination and hemagglutination-inhibition with arthropod-borne viruses	Clarke DH	1958	AM J TROP MED HYG	3,81%
Dengue and dengue hemorrhagic fever	Rigau-Pérez	1998	LANCET	3,66%
The global distribution and burden of dengue	Bhatt S	2013	NATURE	3,59%
A ligand-binding pocket in the dengue virus envelope glycoprotein	Modis Y	2003	P NATL ACAD SCI	3,47%
Research on dengue during World War II	Sabin AB	1952	AM J TROP MED HYG	3,29%
Origins of dengue type 2 viruses associated with increased pathogenicity in the Americas	Rico-Hesse R	1997	VIROLOGY	3,23%
Structure of dengue virus: implications for flavivirus organization, maturation, and fusion	Kuhn RJ	2002	CELL	3,21%
The dengue viruses	Henchal EA	1990	CLIN MICROBIOL REV	3,16%
Dengue: a continuing global threat	Guzman MG	2010	NATURE	3,09%
Dengue: an escalating problem	Gibbons RV	2002	BRIT MED J	3,09%

**Fig. 3:**
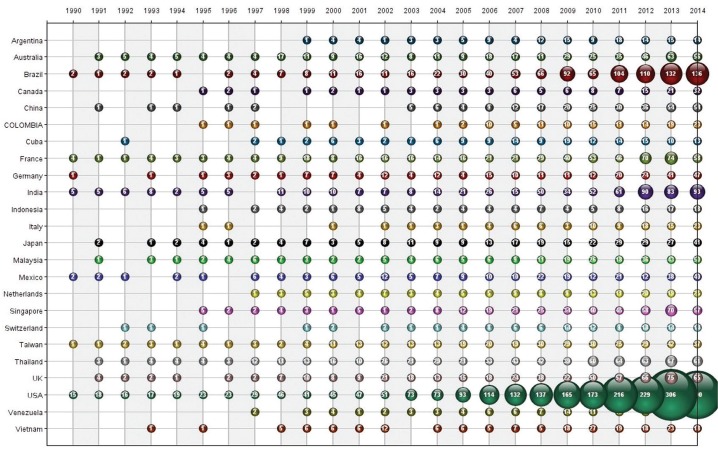
: countries’ dengue scientific publications over time (1990-2014).

USA (30.69%), Brazil (11.98%), Thailand (8.34%), India (8.06%) and France (7.05%) were the top five countries publishing scientific papers on dengue. Countries’ data were retrieved from author’s organisational affiliations. Since articles frequently have co-authors, the sum of countries article’s records is higher than the sum of articles in the database. Overall, the increase in the annual growth of publications for dengue became more significant from 2006 mostly due to USA publications (Fig. 3), which surpassed the symbolic barrier of 100 articles per annum in that same year. From 2005 to 2014, USA publications grew 312%, reaching a peak of 306 articles in 2013.

To a certain extent, this growth could be related with USA military operations in dengue endemic areas, which created demands for dengue treatment, immunisation and vector control. Dengue has been a problem to USA military operations since the Spanish-American War in 1898, frequently occurring before and during World War II in Asia and South Pacific regions. From 1960s to 1990s, dengue cases were reported in USA military operations in Vietnam, Philippines, Somalia and Haiti. The increase in dengue incidence worldwide suggests it will remain a problem for USA military operations until an effective vaccine is licensed (Gibbons et al. 2012). As we will see further, USA military organisations were among the most relevant research organisations on dengue. Further information on this matter can be found in the work of Beaumier et al. (2013) on USA military tropical medicine regarding dengue and other tropical infectious diseases.

An evaluation of research areas (RAs) indicated ‘Tropical Medicine’ (20.41%) and ‘Virology’ (18.46%) as the most frequent themes of research in the dataset, followed by ‘Infectious Diseases’ (17.87%), ‘Public, Environmental and Occupational Health’ (15.43%) and ‘Parasitology’ (11.07%). RAs are assigned to each article at the time of indexing in WoS and reflect the subject areas addressed by them. One must bear in mind that RAs are article-based and an article can be assigned to one or more RA. There are currently 151 RAs in the WoS, from five macro areas: Life Sciences and Biomedicine; Physical Sciences; Technology; Arts & Humanities; and Social Sciences.

**Fig. 4:**
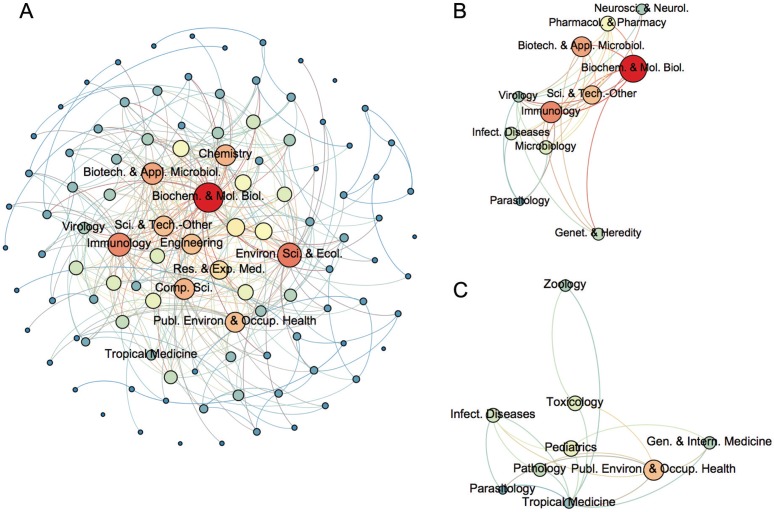
: co-occurrence network of research areas (2000-2014). *Each node represents a research area (RA) and a connection between two RAs indicated they have occurred together in the same article. The size of the nodes indicates their degree centrality in the network. Bigger nodes are the most central. (A) Whole network. The 10 most central areas, according to their degree centrality, have their names indicated; (B) Virology ego-network; (C) Tropical Medicine ego-network.

A network of RAs was built according to their co-occurrence in the same article, providing an insight into the knowledge system involved in dengue research (Fig. 4). Each node represents a RA and a connection between two RAs indicated they have occurred together in the same article. The construction of this network was based on the conception of science maps, which are representations of scientific areas in which the elements are the topics or themes of the mapped area (Noyons 2001). The RAs were positioned on the map so that others frequently occurring together in the same article were located nearby, while those who were less similar were positioned in remote locations. The purpose of this representation was to allow the user to explore the relationships between the areas. The RAs co-occurring more frequently with other RAs have many connections in the network and consequently have high degree centrality, meaning that they are involved in most articles related to dengue.

One hundred and five RAs were involved in the network, characterising the multidisciplinarity of dengue research (Fig. 4A). As depicted, ‘Biochemistry & Molecular Biology’, ‘Environmental Sciences & Ecology’, ‘Immunology’, ‘Biotechnology & Applied Microbiology’ and ‘Chemistry’ were the top five central areas of research in dengue, respectively. These RAs can be considered relevant research subjects in dengue research over the past 15 years. ‘Biochemistry & Molecular Biology’ had the highest degree centrality in the network, indicating it was associated to most RAs in articles on dengue. This result is in line with the worldwide expansion of health biotechnology that has been occurring in both developed and developing countries (Thorsteinsdóttir et al. 2004).

Fig. 4B-C shows the ego-networks of ‘Virology’ and ‘Tropical Medicine’, respectively, the most frequent RAs in the dataset. Despite occurring more often in the whole set of articles, ‘Virology’ and ‘Tropical Medicine’ were not frequently related to other areas and had fairly distinct networks. ‘Virology’ was related to ‘Biotechnology & Molecular Biology’, ‘Biotechnology & Applied Microbiology’ and ‘Immunology’ (Fig. 4B). ‘Tropical Medicine’ was associated to ‘Public, Environmental & Occupational Health’, ‘Toxicology’ and ‘General & Internal Medicine’ (Fig. 4C). ‘Infectious Diseases’ and ‘Parasitology’ were the only RAs in common between these two ego-networks. Overall, the networks suggested that publications in ‘Virology’ were mostly based on biomedical and basic research, while the ones in ‘Tropical Medicine’ were more related to public health and clinical research.

**Fig. 5:**
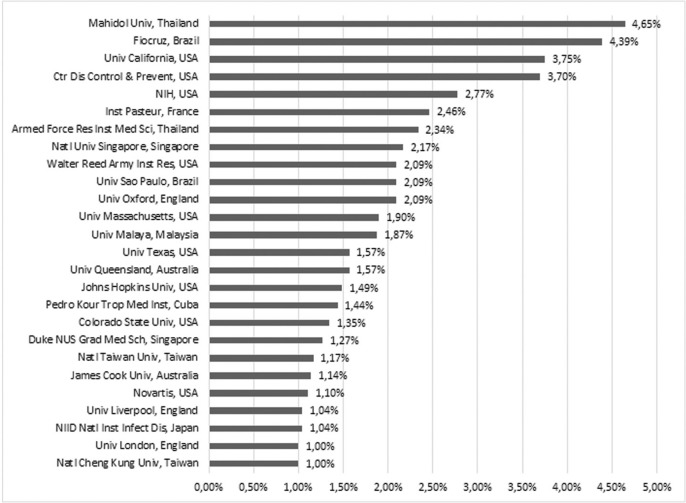
: most frequent research organisations (1990-2014).

Fig. 5 shows the most important research organisations related to dengue according to the percentage of scientific publications. Mahidol University, from Thailand, and Fiocruz, from Brazil, were the two most scientifically productive research organisations on this subject with respectively 4.65% and 4.39% of total world publications. These organisations were followed closely by the University of California, the Centers for Disease Control and Prevention (CDC) and the National Institutes of Health (NIH): all three from USA.

**Fig. 6:**
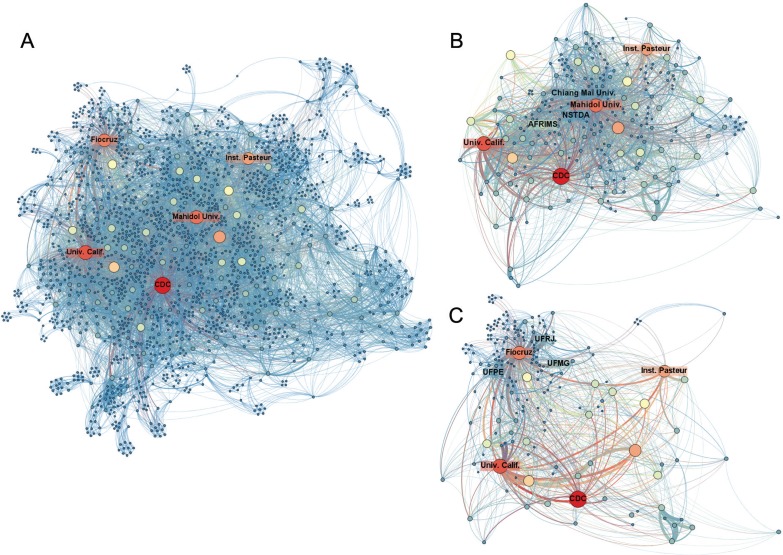
: organisational network of research in dengue (2000-2014). *Links between organisations were mapped according to the affiliations of the authors of scientific papers. Each node represents one organisation and two organisations were considered connected if their authors shared the authorship of a paper. The thickness of the links indicates the frequency of collaboration between two nodes. The node size indicates its degree centrality: bigger nodes are more central. For visualisation purposes, only the largest component of the network is shown. The top five central organisations are indicated. (A) Whole network of organisations involved in dengue research; (B) Mahidol University ego-network; (C) Fiocruz ego-network.

The global organisational network of research in dengue is shown in Fig. 6. Links between organisations were mapped according to the affiliations of the authors of scientific papers. Each node represents one organisation and two organisations were considered connected if their authors shared the authorship of a paper. The size of the network per se indicates the highly collaborative environment for dengue research, which involved 5,296 organisations. CDC was the most central organisation, involved in collaborations with 410 other institutions in the past 15 years. CDC is best known as the USA public health institute and, in spite of not being properly a research organisation, it has important R&D centres, facilities and laboratories carrying significant research on tropical infectious diseases as, e.g., its Division of Vector-Borne Diseases. The CDC Dengue Branch, located in San Juan, Puerto Rico, also provides global leadership in dengue risk assessment, research and effective public health practices. It is important to mention that degree centrality is a proxy for collaboration and not always a measure of the volume of publications. CDC was not the most productive organisation but as it collaborates with many others, it usually has access to different pools of information, leading knowledge exchange and, in consequence, is more likely to be associated with innovative activities (Bell 2005).

Other organisations such as the University of California (USA), Mahidol University (Thailand), Fiocruz (Brazil) and Institute Pasteur (France) were also highly central (Fig. 6A). Being central in this network means that they could have helped to both disseminate knowledge and facilitate access to resources and research opportunities, reducing the network vulnerability. As central organisations, they probably had a vital role in maintaining the connection between the overall research network and in ensuring that less well-connected or peripheral organisations gain access to new knowledge and information. Central organisations are potential sources of information on technology trends, new partnerships and alliances to guide strategic decisions on investments.

It’s important to mention that high centrality is a measure of collaboration and knowledge access, and does not necessarily imply in high scientific productivity. Although Mahidol University and Fiocruz published more articles than CDC, they exhibited a less collaborative behavior, and therefore had smaller degree centralities. Nonetheless, Mahidol University and Fiocruz are important representatives of research organisations from endemic countries of upper-middle-income economies that are currently hosting Phase 2 studies for a new dengue vaccine (Whitehead 2015). Their ego-networks could assist in the understanding of important aspects of their collaboration patterns that can support strategic decisions.

Mahidol University is a Thai multi-disciplinary and research-led institution with key competencies in the arts, medicine and science. Fiocruz is a science and technology organisation of the Brazilian Ministry of Health and one of the most prominent public health institutions in Latin America. The ego-networks of these two organisations (Fig. 6B-C, respectively) showed collaboration with various research institutions in common, such as the University of California, CDC and Institute Pasteur. Fiocruz collaborated with 317 institutions in the period evaluated, mostly with Brazilian universities, such as the Federal Universities of Rio de Janeiro (UFRJ), Pernambuco (UFPE) and Minas Gerais (UFMG). Mahidol University collaborated with 328 organisation in the same period, with a more frequent cooperation with government organisations, such as the National Science Technology and Development Agency (NSTDA) and the Armed Forces Research Institute of Medical Sciences (AFRIMS). AFRIMS is a joint medical research laboratory in Thailand of USA and Royal Thai Army Medical Departments focused on a large number of tropical infectious diseases. Nearly all AFRIMS research efforts are collaborations with local or regional partners (Brown & Nitayaphan 2011).

Being the two most scientifically productive organisations in dengue research, located in dengue-endemic countries, it would be expected that cooperation between Mahidol University and Fiocruz would be commonplace. However, over the period 2000-2014, no papers were co-authored by these organisations. Essentially, there is a low level of coordination and complementarity in the actions of these two public institutions. It is natural for researchers to have a greater propensity to collaborate when working in the same region because the exchange of knowledge becomes easier (Abramovsky & Simpson 2011), but a good strategy for creating a base for innovation requires a network of institutions of excellence with great knowledge intensity. A strategic alliance between them could expand the R&D process in dengue and make it more effective.

This paper has aimed to contribute to upcoming debates, decision-making and planning on dengue R&D and public health strategies by offering to the scientific community, policy makers and public health practitioners a mapping of the dengue scientific landscape worldwide. We extend methodologies currently deployed and combine bibliometrics and SNA techniques to identify the basis of knowledge production in the area and key publications, most important countries, main research areas, key players and their connections. While bibliometric methods make clear which publication, journal, research area or institution attracted most attention by measuring one-dimensional indicators, SNA shows the linkage of these elements in a knowledge domain.

The results showed a significant increase in dengue publications over time - mostly due to the rise of USA publications -, the predominance of research in tropical medicine, virology and infectious diseases, and USA, Brazil and Thailand as the most relevant countries. Research related to biochemistry and molecular biology was largely used to build the knowledge base in the area. The results also depict Mahidol University, Fiocruz and University of California as the main research organisations working in dengue research, while CDC is the most collaborative and therefore most central. The identification of core organisations can provide basis for engaging in dialogue with researchers and institutions actively involved in dengue research and for monitoring their scientific advances over the long term. These organisations can also act as sources of information on technology trends, help identify potential partners for cooperation and reference strategic decisions on public health investments.

These findings, along with other R&D indicators, can strengthen a knowledge platform to inform future policy, planning and funding decisions and also give useful directions to the researchers and institutions working in the area.
